# A case of tuberculosis meningitis after allogeneic hematopoietic stem cell transplantation for relapsed Acute Myeloid Leukemia

**DOI:** 10.1111/tid.13482

**Published:** 2020-10-22

**Authors:** Jinyoung Yang, Sunghyun Moon, Minsuk Kwon, Kyungmin Huh, Chul Won Jung

**Affiliations:** ^1^ Department of Medicine Samsung Medical Center Sungkyunkwan University School of Medicine Seoul Korea; ^2^ Division of Hematology‐Oncology Department of Medicine Samsung Medical Center Sungkyunkwan University School of Medicine Seoul Korea; ^3^ Division of Infectious Diseases Department of Medicine Samsung Medical Center Sungkyunkwan University School of Medicine Seoul Korea

**Keywords:** acute myeloid leukemia, meningitis, miliary TB, stem cell transplantation, tuberculosis

## Abstract

We report a case of tuberculosis (TB) meningitis after allogeneic hematopoietic stem cell transplantation (HSCT) for relapsed acute myeloid leukemia. The patient was 52‐year‐old woman who had relapsed leukemia with a remission duration of 7 months, and she received re‐induction with consolidation, allogeneic HSCT. After 4 days of engraftment, she had headache with fever and cerebrospinal fluid (CSF) analysis presented increased intracerebral pressure, white blood cell counts with dominant neutrophils, elevated glucose and protein level. Brain imaging showed diffuse leptomeningeal enhancement with scattered miliary TB lesions suggesting disseminated TB disease. Mycobacterium tuberculosis was detected in CSF and sputum anti‐TB medication was started. She was IGRA positive before transplantation but did not receive treatment for LTBI prior or during the transplant. Unfortunately, she expired because of intracerebral hemorrhage. TB meningitis is a rare but important complication of HSCT as it can cause serious neurologic sequelae, even death. So in transplant recipients having high risk of TB reactivation, LTBI treatment is recommended before or along with transplantation. If latent TB is not treated, vigilant suspicion and early diagnosis of TB meningitis could improve the transplant outcome.

## 1 INTRODUCTION

Central nervous system (CNS) involvement of tuberculosis (TB) is quite infrequent but can cause serious neurologic damage and even death. Management of TB after allogeneic hematopoietic stem cell transplantation (HSCT) is challenging as a result of severely compromised cell‐mediated immunity in transplant recipients, drug‐drug interactions with immunosuppressants, and hepatotoxicity of anti‐tuberculosis medications. We report an interesting case of TB meningo‐encephalitis with pulmonary TB that developed early after allogeneic HSCT in a patient with acute myeloid leukemia (AML) in the second complete remission (CR).

## 2 CASE DESCRIPTION

A 52‐year‐old female patient was diagnosed with AML during treatment for hypothyroidism. The peripheral blood blast count was 77%, bone marrow biopsy showed normocellular marrow with increased blasts (45.4%), and a *CEBPA* gene double allelic mutation was detected (c.158del [p.Gly53Alafs*107] and c.917_934del [p.Arg306_Gln311del]). Immunophenotype of the blasts was cMPO + CD117+CD13 + CD33+CD7+. Cytogenetics was normal (46,XX), and *FLT3‐ITD* and *NPM1* mutations were not found. The patient achieved complete remission after idarubicin plus cytarabine induction, and she received three cycles of high‐dose cytarabine plus idarubicin consolidation. However, AML relapsed without clonal evolution after 7 months. In June 2019, she received re‐induction with CLAG‐M (cladribine, cytarabine and mitoxantrone) with granulocyte‐colony stimulating factor (G‐CSF) and achieved the second CR. After one cycle of high‐dose cytarabine consolidation, she received allogeneic stem cell transplantation from her HLA 8/8 matched brother. As a screening test before stem cell transplantation, she underwent Quantiferon‐TB Gold Plus (IGRA Qiagen). The result was positive (TB1 Ag minus Nil: 1.370iu/ml, TB2 Ag minus Nil: 1.78IU/mL), but as the chest x‐ray was normal, HSCT proceeded as scheduled without treatment of the latent tuberculosis infection (LTBI). After fludarabine combined with busulfan and antithymocyte globulin (ATG) conditioning 3.87 × 10^6^/kg CD34(+) cells were infused on the 15th of October 2019, engraftment was verified 2 weeks later.

**FIGURE 1:**
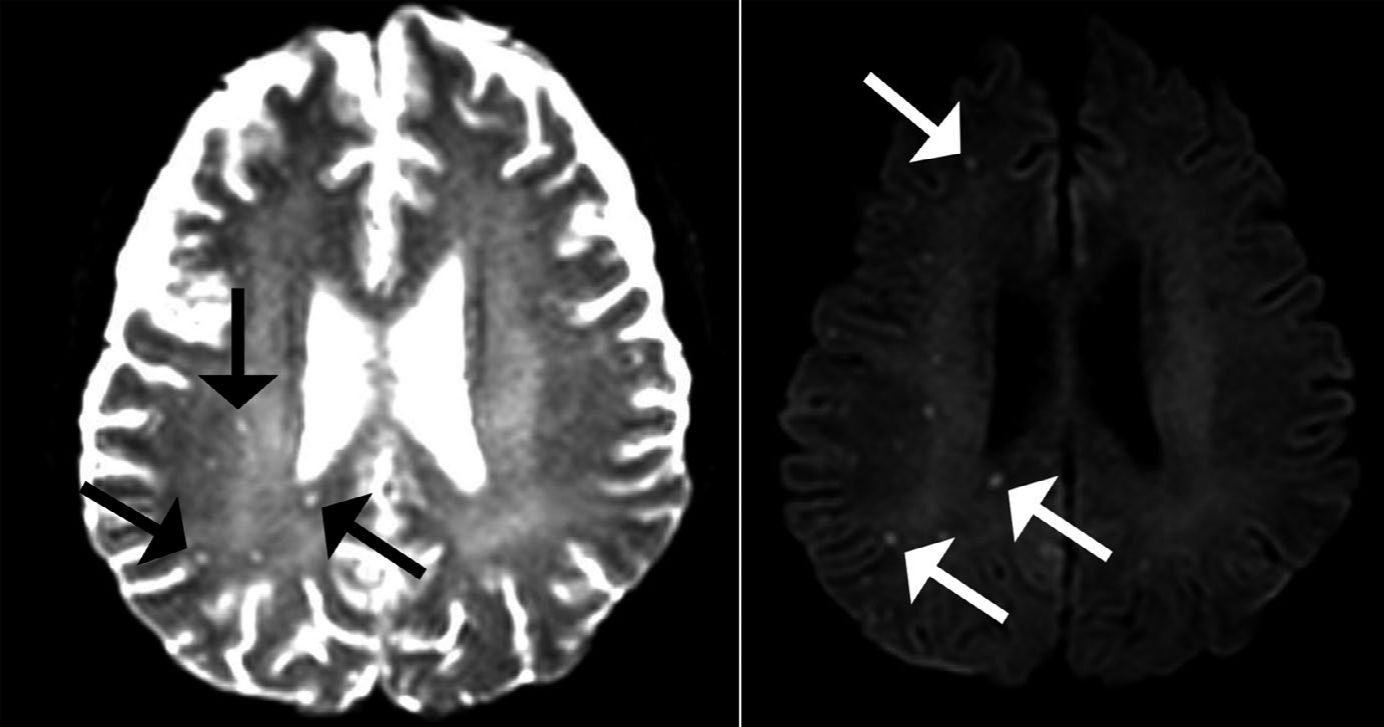
Axial diffusion‐weighted magnetic resonance imaging of this patient showed diffuse abnormal leptomeningeal enhancement and scattered, tiny diffusion restricted foci in both cerebral hemispheres and the cerebellar vermis. Black and white arrows indicate tiny, scattered enhancing lesions, which suggested tuberculosis infection

Three days after conditioning, the patient developed a fever up to 38.3℃. She received empirical antibiotics (piperacillin/tazobactam and vancomycin). By that time, fever was subsided the following day and even if IGRA was positive, the risk of TB development was low and we should considered more common causes of fever immediately after conditioning such as bacterial infection or drug fever. On 10 days after conditioning, *Candida glabrata* was isolated at the site of the Hickmann catheter so anti‐fungal agent was added. Chest PA continued to show no active lung lesion. However, the high fever persisted despite resolution of candidemia. On the 2nd of November 2019, 25 days after HSCT, she complained of a severe headache, so a cerebrospinal fluid (CSF) study was performed by lumbar puncture. Opening pressure was 44 mmH_2_O (normal range: 5‐20 mmH_2_O), white blood cell (WBC) 8/μL (normal range: <5/μL; while WBC from peripheral blood was 350/μL), neutrophils 89%, glucose 71 mg/dL (normal range: 45‐60 mg/dL), protein 44.2 mg/dL (normal range: 20‐40 mg/dL), and adenosine deaminase (ADA) was 3.4 IU/L (normal range: 0‐10 IU/L). Brain non‐contrast computed tomography (CT) grossly showed no newly developed significant focal lesion, but contrast magnetic resonance imaging (MRI) showed diffuse leptomeningeal enhancement with microabscesses scattered in the brain, suggesting a tuberculosis infection (Figure 1).

**FIGURE 2:**
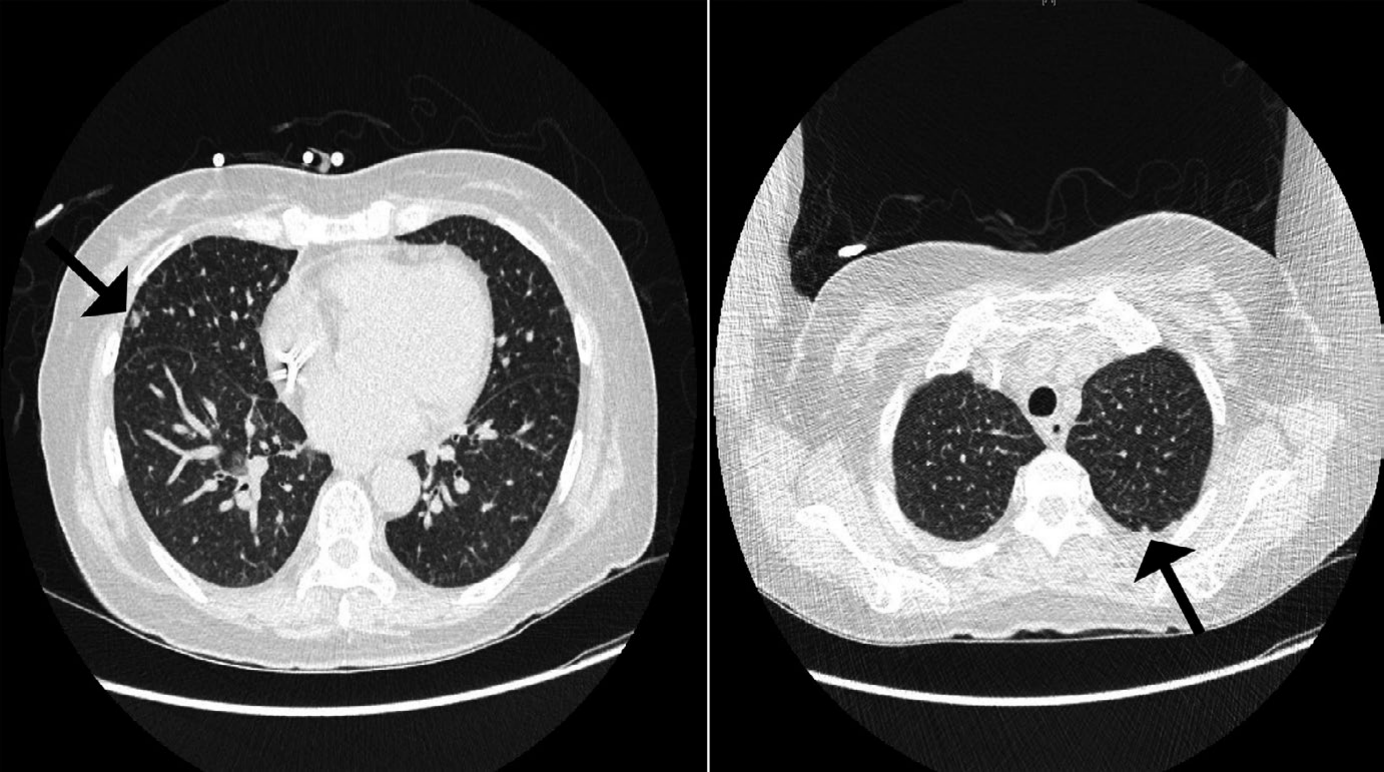
Chest non‐contrast computed tomography showed tiny nodules in both lungs. Arrows indicate tiny nodules in the right middle lobe and left upper lobe of the lungs, suggestive of disseminated tuberculosis infection

The patient became drowsy and showed respiratory depression, and she was transferred to the intensive care unit for mechanical ventilation. For intracranial pressure (ICP) control, mannitol and dexamethasone were used. Twenty‐eight days after HSCT, sputum Xpert MTB/RIF (Cepheld) was positive, sputum, CSF culture, and MTB PCR of the CSF were also positive, so TB infection was confirmed. Table 1 represents the drug susceptibility test of *M tuberculosis* isolated from cerebrospinal fluid. Chest x‐ray showed increased opacity in both lungs, and chest CT demonstrated two tiny nodules in the superior segment of the LLL and diffuse interlobular septal thickening in both lungs (Figure 2). Although the radiologists suggested that the nodules were too small to be characterized, pulmonary TB was strongly suspected. All findings were evaluated together, and she was diagnosed with TB meningo‐encephalitis along with pulmonary miliary TB. There was no evidence of TB involvement in the bone marrow. She had not been treated for TB before and had no history of exposure to TB, but she received Bacillus Calmette‐Guérin (BCG) vaccination in childhood according to national immunization program.

**TABLE 1:** Drug‐susceptibility test result of *M. tuberculosis* isolate from cerebrospinal fluid culture

Drug	Test concentration (μg/ml)	Absolute concentration method	Result
Isoniazid	0.2	—^a^	S^b^
Isoniazid	1.0	—	S
Rifampin	40	—	S
Streptomycin	10	—	S
Ethambutol	2.0	—	S
Kanamycin	30	—	S
Capreomycin	40	—	S
Prothionamide	40	—	S
Cycloserine	30	—	S
Para‐amino salicylic acid	1.0	—	S
Ofloxacin	4.0	—	S
Moxifloxacin	1.0	—	S
Amikacin	30	—	S
Levofloxacin	2.0	—	S
Rifabutin	20	—	S
Linezolid	2.0	—	S
Pyrazinamidase test		—	S

^a^ No growth

^b^ S: Susceptible; Drug‐susceptibility test using absolute concentration method with Löwenstein‐Jensen medium was performed to *M tuberculosis* isolated from cerebrospinal fluid.

She received combination anti‐TB medications of isoniazid (INH) 300 mg PO once daily, linezolid (LZD) 600 mg IV q24hr, moxifloxacin (MXF) 400mg IV q24hr, ethambutol (EMB) 1200 mg PO once daily, and amikacin (AMK) 900 mg IV q24hr. Rifampin (RIF) was not used because of the patient's abnormal liver function (total bilirubin 4.8 mg/dL, AST 163 U/L, and ALT 37 U/L) and, in many studies, it seldom reached CSF concentrations exceeding minimum inhibitory concentration (MIC) of TB^1^ so we selected INH which had excellent CNS penetration. Also she was immunocompromised patient and had severe infection so rapid bacteriocidal effect of INH against mycobacterium was needed. We excluded pyrazinamide (PZA) for the first‐line regimen because liver toxicity of PZA is higher than INH or RIF. Also in principle, (a) 9 months of INH, RIF with EMB or (b) prolonged treatment duration of RIF, EMB with FQ are the first‐choice options in pre‐existing liver disease.^2^ But in this case, we chose the regimen conservatively because of two reasons: hyperbilirubinemia was getting worse and the risk of drug‐drug interaction should have been minimized right after transplantation.

**FIGURE 3:**
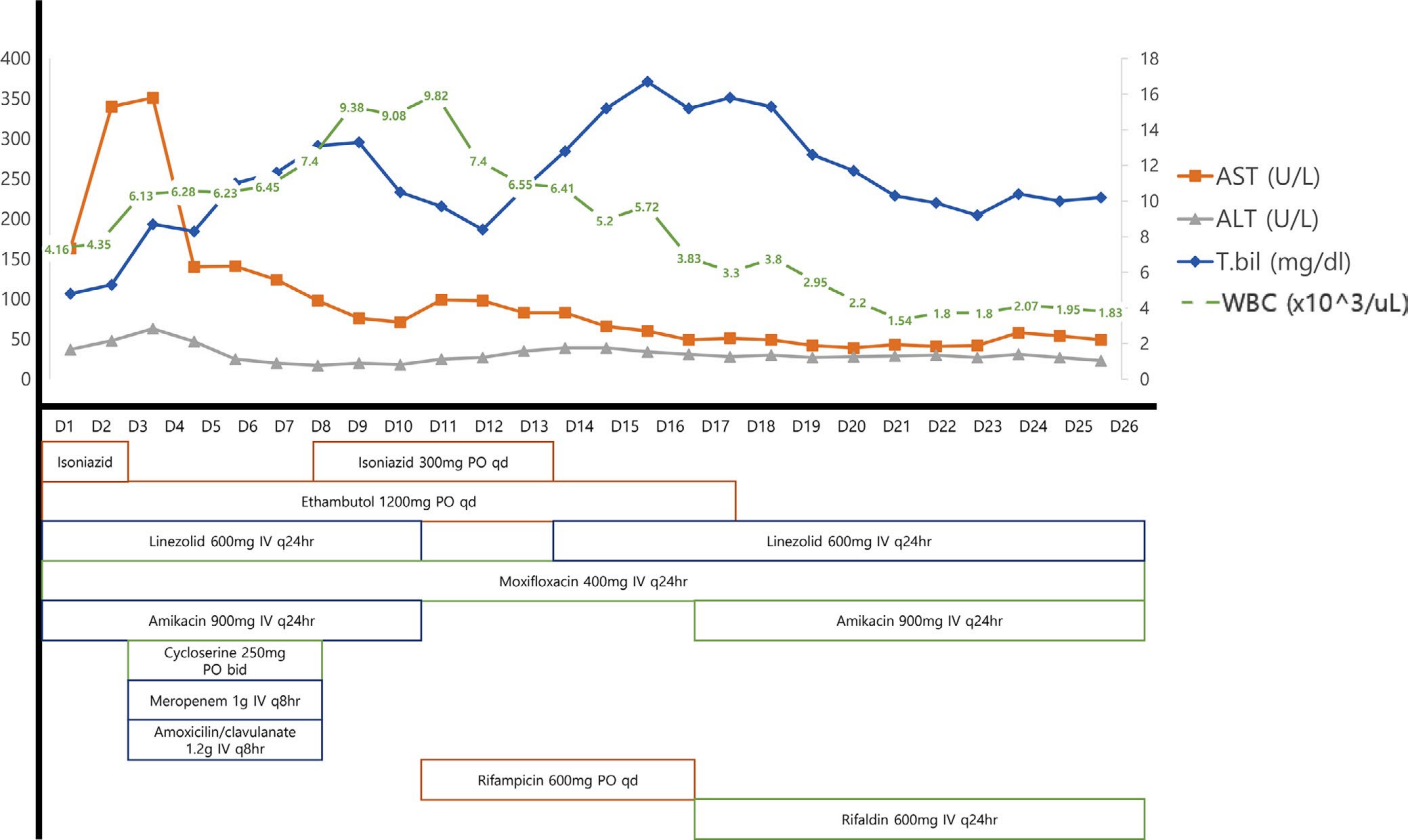
Transition of anti‐tuberculosis medication regimens is showed with changes in liver enzymes, total bilirubin, and white blood cell counts

Two days after starting anti‐TB medication, bilirubin increased to 8.7 mg/dL, AST to 351 U/L, and ALT to 63 U/L. Therefore, INH was discontinued, and cycloserine, meropenem and amoxicillin/clavulanic acid were added to achieve the synergistic effect of clavulanic acid and carbapenem. Seven days after starting anti‐TB medication, liver enzymes normalized, so the first regimen including INH was resumed. At 10 days after initiation of anti‐TB medication, RIF was added, and LZD and AMK were discontinued. However, progressive hyperbilirubinemia led to again changing INH to LZD on day 13. Two days later, GI bleeding occurred. Therefore, oral RIF was changed to IV rifaldin, and EMB was switched to AMK IV (Figure 3).

Thereafter, progression of pancytopenia was noted, and LZD was changed to INH and EMB. At day 27, rifaldin was discontinued because a drug fever was suspected.

Brain MRI on day 20 of anti‐TB medication showed decreased abnormal leptomeningeal enhancement, but it also demonstrated an increased number of enhancing lesions scattered in both cerebral hemispheres with multifocal hemorrhage. Chest CT showed patchy consolidation along the central bronchovascular bundles in both lungs. However, the radiologists suggested that these findings were more suggestive of fungal or other bacterial infection rather than TB. A follow‐up CSF analysis was not performed.

Therefore, according to the second‐line regimen of the WHO anti‐TB medication guideline, we decided to maintain INH, EMB, MXF, and AMK, which would have been continued until 20 months after negative conversion of the AFB stain.

For management of TB meningo‐encephalitis, we administered dexamethasone 10mg four times a day for 6 days and then tapered over a period of 2 months. In addition, mannitol and glycerin were given to control increased ICP.

Unfortunately, although the TB meningo‐encephalitis and pulmonary miliary TB were well controlled, the patient expired 115 days after stem cell transplantation because of an intracerebral hemorrhage that developed suddenly owing to prolonged thrombocytopenia.

## 3 DISCUSSION

In 2018, the incidence of new TB cases in South Korea was 51.5 per 100 000 population, quite high compared to the global incidence of TB of 132 per 100 000 according to the WHO global tuberculosis report from 2019.^3^ However, even in countries where TB is common, the incidence of TB after HSCT is extremely low. In previously reported data from our HSCT cohort from 1996 to 2003,^4^ the incidence of TB after HSCT was 3.1% (9 of 295 allo‐ and auto‐HSCT). Among the nine cases of TB after HSCT, there were 7 cases of pulmonary, 1 case of pericardium, and 1 case of pulmonary with spine. No cases of meningitis were identified. In Pakistan, where the TB prevalence is higher than our country, the incidence of TB after HSCT was 2.6% (4 of 154 all HSCT) from 2001 to 2006.^5^

TB meningo‐encephalitis accounts for 1% of all tuberculosis infections. In one study of solid organ transplantation, TB occurred in 0.48% (21 of 4388) of patients, and there was only 1 case of TB meningo‐encephalitis and 2 cases of disseminated TB.^6^ Another study showed that TB developed in 1.58% after solid organ transplantation (SOT) and 1.02% after HSCT.^7^ Considering the lower number of transplantations of SCT compared to solid organ, it is very rare to experience TB CNS infection after HSCT. However, transplant recipients with TB CNS infections have a high mortality and can suffer serious neurologic sequelae, so early diagnosis and treatment of TB meningitis are clinically very important.

The definitive diagnosis of TB meningitis depends on detecting mycobacterium in cerebrospinal fluid by culture. MTB PCR and radiologic methods are useful to differentiate TB from other infections and to determine the extent of the disease. Detecting the evidence of TB in other organs is also helpful because TB CNS as a form of disseminated tuberculosis infection is common. In the CSF analysis, increased WBC (100‐1000 cells/mL), elevated lymphocyte count, low glucose (<50 or 30 mg/dL or ratio of CSF to blood glucose < 0.4‐0.5), increased protein levels (150‐500 mg/dL), and elevated ADA level (more than 15.5 U/L^9^) are common features of TB meningo‐encephalitis. Although the burden of mycobacterium in the CSF is usually relatively small, repetition of lumbar punctures and sufficient volume of CSF are important to document mycobacterial involvement by culture and molecular study.

The principles of treatment for TB meningitis are the same as those for pulmonary TB, but the duration of medication is longer. In the first 2 months, a combination of INH, RIF, PZA, and EMB is recommended, and if there is no resistance, a combination of INH and RIF is administered for 7 to 10 months after induction treatment. However, if second‐line regimens are needed because of resistance or intolerance, cycloserine, streptomycin, AMK/kanamycin, and levofloxacin/MXF can be used. Among them, MXF was reported to have an excellent penetration rate through the blood‐brain barrier.

In solid organ transplantation, the American Society of Transplantation recommends that all cases of LTBI be treated.^8^ In HSCT, the Center of International Blood and Marrow Transplant Research and Centers for Disease Control and Prevention recommend LTBI treatment in transplant recipients (a) who have been exposed to an individual with active, infectious pulmonary or laryngeal TB, (b) having a positive TST result regardless of prior BCG vaccination without previous treatment and no evidence of active TB disease, and (c) with a positive IGRA result without previous treatment and no evidence of active TB.^9^

Clinicians must also consider drug‐drug interactions of anti‐TB medication in transplant recipients. HSCT recipients usually receive immunosuppressants and prophylactic antibiotics, so there could be toxicities and drug interactions that would make it difficult to interpret laboratory abnormalities.

Generally, it is difficult to delay HSCT because more than 3 months of LTBI treatment would increase the relapse risk of the underlying diseases.

In the present case, the patient was IGRA positive at the screening test before transplantation, but as there was no active lung lesion on chest radiographs, we did not treat LTBI according to our institutional protocol. Furthermore, we could not delay stem cell transplantation for LTBI treatment, considering the relatively short duration of remission of 7 months. In our center, 1187 transplant recipients underwent testing for IGRA from February 2014 to March 2020. IGRA was positive in 225 (18.9%) patients, and the number of patients who were documented to have active TB after transplantation was 12 (5.3%). Among the 12 cases (10 patients were allogeneic and 2 were autologous SCT) of documented active TB after transplantation, there were 7 cases of pulmonary TB, 3 of TB pleurisy, 1 of TB lymphadenitis, and the present case was the only one confirmed to have TB meningitis. Two other patients were suspected to have TB meningitis, an incidence of 0.25%. The patient in the present case and one of the suspected TB meningitis patients expired, underlining the high mortality rate of TB meningitis.

Another study from Korea showed IGRA positivity in 40 of 224 (16.4%) HSCT recipients,^10^ and the authors reported that only one patient developed active TB (2.5%). As the prevalence of LTBI and the reactivation rate of TB are not low, IGRA should be a routine screening test before stem cell transplantation in endemic countries.

Therefore, our experience supports that LTBI treatment before or concurrent with HSCT should be recommended for patients who test positive for IGRA.

## AUTHOR CONTRIBUTIONS

Investigation: Yang JY, Moon SH. Supervision: Kwon MS, Huh KM, Jung CW. Writing – original draft: Yang JY. Writing – review & editing: Yang JY, Kwon MS, Huh KM, Jung CW.

## DISCLOSURE

The authors have no potential conflicts of interest to disclose.
